# Video Abstracts in Research

**DOI:** 10.2196/64221

**Published:** 2024-11-04

**Authors:** Sophie Nachman, Esteban Ortiz-Prado, Joseph D Tucker

**Affiliations:** 1 Institute for Global Health and Infectious Diseases Chapel Hill, NC United States; 2 UNC Gillings School of Global Public Health Chapel Hill, NC United States; 3 One Health Research Group at Universidad de las Américas Universidad de las Américas Quito Ecuador; 4 Clinical Research Department London School of Hygiene and Tropical Medicine London United Kingdom

**Keywords:** video abstract, abstract, dissemination, public engagement, online, videos, public audience, communication, infographics, health literacy, patient education, public health

## Abstract

Video abstracts can be useful in health research. A video abstract provides key messages about a research article and can increase public engagement, spark conversations, and may increase academic attention. A growing number of open source software programs make it easier to develop a video abstract. This viewpoint provides practical tips for creating a video abstract for health research.

## Introduction

Video abstracts can breathe life into health research studies, adding a human dimension to what would otherwise be a dry text. A video abstract is a brief video that provides several key messages about a research article. Video abstracts catalyze public engagement, increase the number of manuscript views, and may increase manuscript citations [1,2]. A growing open science movement provides a strong rationale for developing a video abstract as part of a health research manuscript. Video abstracts provide an opportunity for research participants to codevelop dissemination materials and tailor dissemination for patients, policymakers, and others [3]. Video abstracts in health research are often more appropriate for public audiences compared to text manuscripts [1]. Communicating scientific research in an easy-to-understand way is crucial to improve shared decision-making, trust in health institutions, and health literacy. Many medical journals not only allow video abstracts but encourage them to broaden research impact.

Despite the compelling reasons to develop video abstracts, most health researchers have never developed one. Many researchers are not familiar with video creation, lack open source resources, and have limited training in public engagement. The process of developing a video can be challenging, even among researchers who have communications support. Many online resources for creating videos are paywalled or require software. The purpose of this viewpoint is to review practical, open source strategies for developing video abstracts with a focus on expanding research accessibility for a broad public audience.

## Developing a Video Abstract

Several strategies can help researchers develop their first video abstract. Video abstracts should provide the most important points of the research. A strong video abstract uses plain language [4], communicating the message in a way that a broad public audience can easily understand without reading the accompanying text. One study analyzing COVID-19 videos found them easier to understand and more actionable compared to websites and infographics [5]. Consulting health literacy guidelines and evaluation tools such as the Patient Education Materials Assessment Tool [6] or the Centers for Disease Control and Prevention (CDC) Clear Communication Index [7] can help confirm that content is clearly communicated.

## Developing Inclusive Videos

Accessibility guidelines provide specific strategies for reaching a wider public audience that includes diverse individuals. The Web Accessibility Initiative provides many practical tips for creating accessible audio and video content (Multimedia Appendix 1) [8]. For example, adding captions and providing a transcript are important not only for people with limited hearing but also for people with limited English proficiency [8]. Open source software provides initial drafts of captions that can be revised. Additionally, ensure color contrast in the visual design is sufficient and include narration and audio descriptions for the visuals to provide useful context for the audience.

Organizing the whole research team is essential for creating a compelling video abstract (Table 1). Researchers should consult their local libraries (university and local) to find out what video creation resources are available. Video abstracts are an opportunity for cocreation with community partners who are often involved in research as participants, funders, or evaluators but excluded from research dissemination [3]. Since patients or consumers of health research may not have access to the full-text publication, engaging the intended audience in video creation can increase comprehension and relevance. Cocreation has been a valuable strategy for developing relevant video messages related to consumer health, health literacy, and public health interventions [3,9]. When planning your video abstract, consider who in the community may have creative, artistic, or technological skills to contribute. Also consider whose voice is missing from the manuscript and who is best suited to deliver the message to the intended audience. Aiming for a brief duration (less than 10 minutes) is important for keeping the viewer’s attention. Open source teleprompter software can help to facilitate a smooth delivery.

**Table 1:** Considerations for creating video abstracts.

Category	Considerations
Setting	Find a quiet space with good lighting.
Audio	Use an additional microphone to enhance audio quality.
Filming	Use a tripod for steady filming.
Teleprompter software	Use a teleprompter software application to enhance delivery.
Iterate	Solicit and incorporate audience feedback.
Visuals	Use clear, concise visuals or infographics.
Accessibility	Include captions or subtitles; provide a transcript.
Length	Aim for a brief length (less than 10 minutes)
Open source software	Video editing: Shotcut (Meltytech LLC; Windows, Mac, Linux), OpenShot (OpenShot Studios LLC; Windows, Linux, Mac) Graphic design: GIMP (Kimball & Mattis, GNU Image Manipulation Program), Inkscape (Inkscape Project; Windows, Mac, Linux) Audio recording and editing: Audacity (The Audacity Team) Screen recording: OBS Studio (Bailey, Open Broadcaster Software) Animation: Blender (Roosendaal) Storyboarding: Trelby (Gulecha; Windows, Linux), Storyboarder

## Approaches to Video Abstract Creation

There are several approaches to choose from depending on the goals, skills, and resources available to your team (Table 2). A do-it-yourself (DIY) 1-person approach can be executed within a few days by recording yourself (or a team member) using a cell phone, Zoom, or creating a presentation in PowerPoint (Multimedia Appendix 2, point 2a). This approach requires no special equipment and is relatively simple. As a result, this approach can be used to develop a video abstract within a shorter period of time (eg, several hours). However, this method centers on you and requires some basic editing skills. Alternatively, a DIY drawing approach involves making a time-lapse video of drawn images or text, which can effectively tell a story and incorporate creative drawings (Multimedia Appendix 2, point 2b). A time-lapse video requires some basic materials, including a storyboard, whiteboard, markers, video recording device, basic editing software, and an elevated surface [3]. At the same time, creating time-lapse videos requires some sketching ability, in addition to video editing skills. For a more team-based approach, you can use open source tools that are available online to develop a video abstract (Multimedia Appendix 2, point 2c). This approach can easily include subtitles and draw on existing templates but does require familiarity with open source programs. Generally, this requires a person with greater experience in video creation and a longer period of development. Finally, artificial intelligence (AI) video generation can be used to develop a brief video. While there are increasing resources available to support AI video generation (eg, RunwayML for video generation; Mozilla TTS for voice synthesis), this approach does require more technical expertise [10].

**Table 2:** Approaches and methods for creating a video abstract.

Approach and method		Strengths	Weaknesses
**DIY^a^ 1-person approach**			
	Record yourself (or a team member) using a cell phone, Zoom, or PowerPoint	No equipment required Relatively simple Can create a video within days	Centers on you (or a team member) Requires some basic editing skills
	DIY drawing where you make a video of drawn images and text [3]	Easier to tell a story Can incorporate creative drawings Can create a video within days	Some basic equipment needed Requires sketch drawing skills
**Team-based approach**			
	Animation using online tools	Easy to integrate subtitles and text Use of templates and defaults	Requires open source software^b^ for animation May take a longer period of time
	AI^c^ video generation	Easy to develop a brief video of an avatar speaking	Requires open source software^d^ and greater technical expertise

^a^DIY: do-it-yourself.

^b^There are several open source animation programs, including Synfig Studio and Blender.

^c^AI: artificial intelligence.

^d^Open source resources can help with AI video generation (RunwayML) and AI voice synthesis (Mozilla TTS).

## Recommendations for Developing a Video Abstract

We have several recommendations for those interested in developing their first video abstract. First, creating a draft storyboard is an essential first step in developing a video. Storyboards are visual representations of how the video will progress, frame by frame. This can help the researcher create a list of necessary materials and identify who else needs to be involved in the video development. Second, the storyboard can be turned into a detailed script that conveys key points. Third, iteratively recording video and audio can help to develop a compelling video.

## Conclusion

Video abstracts provide a unique opportunity to broaden research dissemination, meaningfully engage communities, and provide a human touch within research. By concisely explaining research in a plain and accessible way, video abstracts can make the results and implications of health research available to a wider audience, including patients and consumers. Once a video abstract is created, this can be included as a URL in supplemental data, uploaded to a video platform or shared drive, or embedded within an online version of a journal. Case studies of video abstracts have demonstrated the impact of brief videos within the larger process of research dissemination (Multimedia Appendix 2). Given the brief duration, a compelling video abstract could also be part of a presubmission inquiry or cover letter. Open access resources and simple strategies can empower researchers to create videos with little or no budget. While research is needed to understand the impact of video abstracts on key research outcomes, there is already a strong evidence base supporting their use. Video abstracts provide an exciting opportunity to spark innovation and creativity in the research process.

## Appendix

Multimedia Appendix 1 Open source resources for video creation.

Multimedia Appendix 2 Case studies of video abstracts.

## Abbreviations

AI: artificial intelligence
CDC: Centers for Disease Control and Prevention
DIY: do-it-yourself
