# Supplementary material for: Video Abstracts in Research
Source: J Med Internet Res. 2024 Nov 4;26:e64221. doi: 10.2196/64221 (PMC11574489; doi:10.2196/64221)
Supplement: Multimedia Appendix 1 [file jmir_v26i1e64221_app1.docx]

**Multimedia Appendix 1.** **Open access resources for video creation.**

1. **University of York “Introduction to Media Editing”:** Provides detailed support on editing videos <https://subjectguides.york.ac.uk/media/video>
2. **Duke Library “Getting Published: Visual and Video Abstracts”**: User-friendly overview of video abstracts <https://guides.mclibrary.duke.edu/gettingpublished/visualabstracts>
3. **Web Accessibility Initiative:** tips for making video accessible <https://www.w3.org/WAI/media/av/>
