# Supplementary material for: Video Abstracts in Research
Source: J Med Internet Res. 2024 Nov 4;26:e64221. doi: 10.2196/64221 (PMC11574489; doi:10.2196/64221)
Supplement: Multimedia Appendix 2 [file jmir_v26i1e64221_app2.docx]

**Multimedia Appendix 2. Case studies of video abstracts.**

*2a. DIY crowdsourced video.* (link [here](https://www.youtube.com/watch?v=WbDpNrV3avg)) This video was solicited by a crowdsourcing open call. The final open call solicited brief videos created by community-based organizations. The final video developed by Danlan was then assessed in a randomized controlled trial and compared to a video developed by a social marketing company. The resulting HIV test uptake was similar between the intervention (crowdsourced) and control (social marketing) arms. The resulting crowdsourced video was viewed 612 times as of 6 September 2024.

Youtube video: <https://www.youtube.com/watch?v=WbDpNrV3avg>

Research study: https://academic.oup.com/cid/article/62/11/1436/1745296

*2b. DIY time-lapse video example.* (link [here](https://www.thelancet.com/doi/story/10.1016/vid.2018.12.18.107578)) This video abstract was developed by an undergraduate summer student with a special interest in communications. The key messages were developed by the research team, then sketched out as a storyboard. This video was used in the pre-submission to *The Lancet Infectious Diseases* where it was ultimately published. *The Lancet* included the video within its Youtube channel, alongside being featured on the SESH Global Youtube site. As of 6 September, the video has been viewed 930 times.

Youtube video: <https://www.youtube.com/watch?v=a7-XsfUV_l0>

Associate article: <https://www.thelancet.com/journals/laninf/article/PIIS1473-3099(18)30556-5/abstract>

*2c. Animated video abstract.* (link [here](https://drive.google.com/file/d/1eiiuu1ihXjuBZremMWBqs06A5liuqHEX/view)) An animated video was developed using open access software by a research assistant and the PI. This was included in the pre-submission to the journal and is now under review.

YouTube video link: https://drive.google.com/file/d/1eiiuu1ihXjuBZremMWBqs06A5liuqHEX/view

Pre-print: https://papers.ssrn.com/sol3/papers.cfm?abstract_id=4670498
